# Per- and Polyfluoroalkyl Substances (PFAS) Neurotoxicity in Sentinel and Non-Traditional Laboratory Model Systems: Potential Utility in Predicting Adverse Outcomes in Human Health

**DOI:** 10.3390/toxics8020042

**Published:** 2020-06-15

**Authors:** Rachel Foguth, Maria S. Sepúlveda, Jason Cannon

**Affiliations:** 1 School of Health Sciences, Purdue University, West Lafayette, IN 47907, USA; rfoguth@purdue.edu; 2 Purdue Institute of Integrative Neuroscience, Purdue University, West Lafayette, IN 47907, USA; 3 Department of Forestry and Natural Resources, Purdue University, West Lafayette, IN 47907, USA; mssepulv@purdue.edu

**Keywords:** per- and polyfluoroalkyl substances, sentinel species, perfluorooctane sulfonate, perfluorooctanoate, perfluorobutane sulfonate

## Abstract

Per- and polyfluoroalkyl substances (PFAS) are a class of chemicals that were widely used in manufacturing and are now present in the environment throughout the world. It is known that various PFAS are quantifiable in human in blood, but potential adverse health outcomes remain unclear. Sentinel and non-traditional model species are useful to study potential toxicity of PFAS in order to understand the relationship between environmental and human health. Here, we present a critical review of studies on the neurotoxicity of PFAS in sentinel and non-traditional laboratory model systems, including *Caenorhabditis elegans* (nematode), *Dugesia japonica* (planarian), *Rana pipiens* (frogs), *Danio rerio* and *Oryzias melastigma* (fish), and *Ursus maritimus* (polar bears). PFAS have been implicated in developmental neurotoxicity in non-traditional and traditional model systems as well as sentinel species, including effects on neurotransmitter levels, especially acetylcholine and its metabolism. However, further research on the mechanisms of toxicity needs to be conducted to determine if these chemicals are affecting organisms in a similar manner. Overall, findings tend to be similar among the various species, but bioaccumulation may vary, which needs to be taken into account in future studies by quantifying target organ concentrations of PFAS to better compare different species. Furthermore, data on the majority of PFAS is lacking in neurotoxicity testing, and additional studies are needed to corroborate findings thus far.

## 1. Introduction

**Figure 1:**
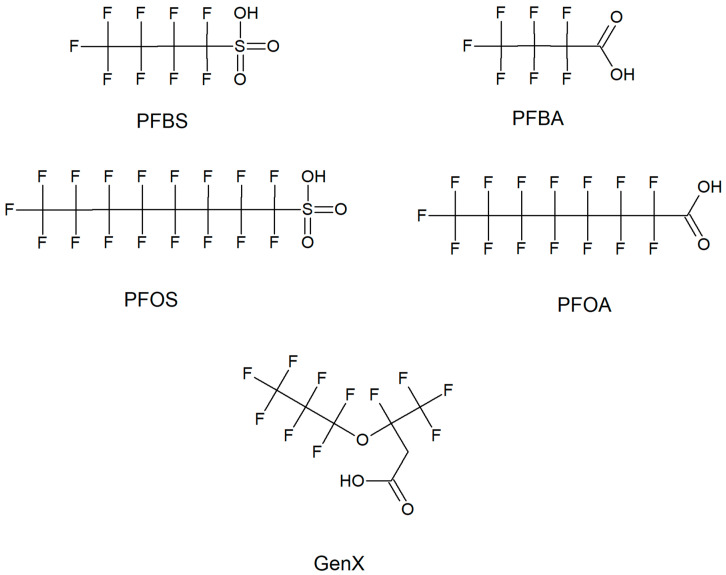
Structures of the representative per- and polyfluoroalkyl substances (PFAS) discussed in this article. Short-chain PFAS are represented by perfluorobutane sulfonate (PFBS) and perfluorobutanoic acid (PFBA). Long-chain PFAS are represented by perflourooctane sulfonate (PFOS) and perfluorooctanoic acid (PFOA). Next generation PFAS are represented by GenX. For a more inclusive view of PFAS structures see Shaw et al., 2019 and Xiao, 2017 [5,6].

Per- and polyfluoroalkyl substances (PFAS) are chemicals that have been used in industry since around the 1950s [1,2]. These compounds were used during manufacturing for common household materials, such as nonstick cookware, food packaging, antistatic agents, anti-stain agents, water repellants, firefighting foams, and hygiene products due to their surfactant properties [2,3]. The longer chain PFAS, with 7 or more carbons (such as perfluorooctanoic acid, PFOA, and perfluorodecanoic acid, PFDA) were first phased out of the United States in 2001 due to findings of toxicity; however, they are still prevalent in the environment due to their strong carbon–fluorine bonds, which makes them extremely stable and persistent, and because they are still used in other parts of the world and can be shipped to the United States (Figure 1) [1,2]. Furthermore, alternatives to the longer chain PFAS are shorter chain PFAS (less than 7 carbons) such as perfluorobutanoic acid (PFBA) and perfluorobutane sulfonate (PFBS) because they are generally believed to bioaccumulate less (Figure 1) [1,4,5,6]. It is worth noting that there are significant caveats to this assumption. In general, far more data are needed to definitively show that shorter chain PFAS accumulate less than longer chain PFAS. This is especially highlighted by evidence of shorter chain (6 carbon) PFAS with long half lives, such as perfluorohexane sulfonate, PFHxS (>5 years) [7]. Furthermore, these compounds still are not metabolized, are prominent in water, including that for drinking, and have largely unknown toxicological effects [1,4]. Furthermore, a novel PFAS, hexafluoropropylene oxide dimer acid (GenX), has been found in both the Cape Fear River and the blood of people who live in the Lower Cape Fear River Basin along with other locations, such as in the Netherlands (Figure 1) [4,8,9,10]. Thus far, research on the neurotoxicity of GenX is limited to the finding that rat brain capillaries had decreased transport activity of P-glycoprotein and breast cancer resistance protein, which are two enzymes that are important for the proper function of the blood–brain barrier, indicating the potential effects of transport of chemicals into and out of the brain [11].

PFAS are now found throughout the world. There are several notable examples of major contaminated sites in the United States such as Wurtsmith Air Force Base in Michigan with water concentrations measuring around 110 µg/L perflourooctane sulfonate (PFOS), 104 µg/L PFHxS, 105 µg/L PFOA, and 5 µg/L perfluorohexanoate (PFHxA), similar to other locations throughout the world [2,12,13,14]. Furthermore, there are measurable levels of PFAS in humans, with the overall mean concentrations in serum from 2016 to 2018 being 2.94 ng/mL PFOS, 2.04 ng/mL PFOA, 1.10 ng/mL PFHxS, and 0.79 ng/mL perflourononanoate (PFNA) [15,16]. Besides just being measured in serum, the half-life of these compounds has been calculated from exposed fluorochemical workers to be around 5.4 years for PFOS, 8.5 years for perfluoroheptane sulfonate (PFHpS), and 3.8 years for PFOA [7]. Unfortunately, there are many more PFAS than those measured in humans thus far, so exposure is not completely understood. Exposure to these compounds is not just problematic for adults, as exposure occurs throughout life. PFAS have been quantified in breastmilk, amniotic fluid, and fetal tissue, including the liver, lung, heart, and brain, umbilical cord blood, and placenta, indicating that infants are being exposed from the very beginning of life [17,18,19,20,21,22]. Studies in model systems such as Cymologous monkeys, rats, and mice have indicated various forms of toxicity; however, the shorter half-lives of PFAS in rodents requires a large exposure to obtain internal doses similar to those in humans. It is also beneficial to determine the effects of chemicals on many different organisms because similarities can indicate toxicity that is more likely to occur in humans. One type of study that can be useful for determining toxicity is by using sentinel species, which are organisms naturally exposed to the chemicals of interest and could indicate toxicity earlier than it is seen in humans. Furthermore, certain types of sentinel species are physiologically very similar to humans, such as polar bears (*Ursus maritimus*). The use of other model systems that are traditionally used in the laboratory, such as zebrafish (*Danio rerio*) and nematodes, could help determine effects that could be further studied in other model species more similar to humans. This article focuses on a review of the current literature that examines the neurotoxicity of PFAS on sentinel and non-traditional model species. Major areas of research on PFAS toxicity include aspects such as hepatotoxicity, cholesterol and lipid distribution, immunotoxicity, and effects on the thyroid [23]. One aspect of PFAS toxicity that is important to study is neurotoxicity. Research has shown that mice exposed to PFOS or PFOA at postnatal day 10 had increased activity at 2 months of age and decreased movement at 4 months of age, indicating potential effects on motor function [24]. Interestingly, mice exposed to PFOS during development had significantly decreased movement after treatment with methamphetamine, potentially indicating effects on reward-based movement [25]. Furthermore, rats exposed to 20 mg/kg PFOS daily for 28 days starting at 2 months had decreased myelination in the brain, also indicating neurotoxicity [26]. Unfortunately, there is still much to understand about the potential neurotoxicity of PFAS. 

## 2. Qualities of Good Sentinel Species

There are specific characteristics that make certain animals good sentinel species. Species that are more susceptible to toxicity after exposure could indicate toxicity to other species, such as humans. For a complete summary of the differences among monitors, indicators, and sentinels, refer to O’Brien, 1993 [27]. Some of the characteristics to consider include body size, sensitivity, physiology, longevity, and latent period [27]. Body size is important because it could affect the accumulation of chemicals along with exposure, with larger surface areas potentially increasing exposure [27]. Sensitivity is another important aspect because a species could be exposed to the same amount of a chemical but not be as sensitive due to differences in the location of accumulation, metabolism, or pathways affected [27,28]. When assessing differences in sensitivity across species, it is important to consider where contaminants accumulate. For example, whales have an important amount of blubber, so they serve as good sentinels for lipophilic contaminants [28]. To relate the effects of compounds, the physiology being studied needs to be similar between humans and the sentinel species being studied, or at least differences need to be accounted for, because differences in physiology could cause differential effects and limit extrapolation to human relevance [27]. The longevity of a sentinel species is important because often exposure needs to occur over a long time to cause measurable effects; however, sometimes it is useful to use a species that has a shorter lifespan because it is easier to see effects that occur during aging, such as degeneration [27]. Species that have longer lives are better for studying effects such as cancer and reproductive effects [28]. The latent period is important in a similar way to longevity because acute exposure to a chemical can cause significantly different effects than chronic exposure, such as that throughout a lifetime [27]. 

There are many other qualities that should be considered when selecting sentinel species. One such quality is the location on the food chain of the species [27,28]. Humans are at the top of the food chain, so it is important to remember that bioaccumulation can occur, leading to increased exposure. Another quality is migratory patterns, which can affect the exposure of sentinel species, such as if birds migrate somewhere, their exposure is significantly less during half the year [27]. As touched on above, the route of exposure and distribution are important due to changes in metabolism and accumulation after exposure in different ways [27]. The last big factor to consider is the utility of captive sentinel or non-traditional model species [27]. While these qualities are important for sentinel species, they also apply to other model systems, including non-traditional model organisms. These organisms can also indicate potential aspects of toxicity due to accumulation or higher sensitivity, similar to sentinel species, and should be utilized in determining the effects of environmental exposures. This could allow for more in-depth studies than collection from the wild, including more mechanistic studies to understand what is occurring on the molecular level and possible ways to mitigate effects. 

## 3. Invertebrate Species Used to Study Neurotoxicity of PFAS

### 3.1. Caenorhabditis elegans

*Caenorhabditis elegans* are nematodes that are very useful model organisms due to their transparent bodies, fully sequenced genome, and the knowledge of the cell fate of each cell at germination. *C. elegans* are commonly found in the wild in places with rotting vegetation or soil rich in microbiota [29]. In the laboratory, *C. elegans* are studied using both liquid and solid broth with *Escherichia coli* present as a food source. The ability to easily maintain these worms in laboratory conditions also means that they can be exposed to specific chemical insults and studied for the effects over their lifespan. They also have a relatively short lifespan, and there are many strains that contain mutations or fluorescent expression that are useful in studying development and toxicity [30]. Many of these traits have made them useful non-traditional model organisms in studying the toxicity of PFAS and have guided other studies to further determine the mechanisms of toxicity. PFAS studied in *C. elegans* neurotoxicity only focuses on PFOS thus far. 

Few studies have measured the amount of PFAS absorbed and retained after exposure in *C. elegans*; however, it has been shown that *C. elegans* accumulate PFOS to 13.06 µg/mg after 1 mg/L PFOS and accumulation increases dose-dependently, while PFBS does not accumulate (Table 1) [30,31]. The toxicity of PFAS has been of much more interest, with the toxicity of PFOS being the most prominent due to its accumulation and high environmental levels. It has been shown many times that PFOS is toxic in a dose-dependent manner [31,32]. The mechanisms of excretion of PFAS in *C. elegans* is currently unknown, which is a major weakeness when comparing toxicity to other systems. The lethal concentration for 50% death (LC_50_) has been calculated to be 4.522 mg/L (9.04 µM) after 24 h, and 1.4 µM or 2.03 mM after 48 h of exposure [31,33,34]. Discrepancies between these studies could be caused by differences in age, where most papers did not note the developmental stage at exposure or differences in the amount of branching. PFOS can be produced in two different ways, one of which produces 65–79% linear PFOS, while the other produces linear PFOS almost exclusively [35]. Research has indicated that the linear versus branched PFOS accumulate in tissue differently and potentially cause different toxicities [35]. The LC_50_ of PFOA was found to be much higher, 22.655 mg/L (54.7 µM), which is consistent with decreased accumulation as well as decreased toxicity [34]. PFBS was also found to be toxic in a dose-dependent manner, with a calculated LC_50_ of 794 µM, which was much higher than that of PFOA, possibly due to less accumulation or faster excretion due to the shorter chain length [31]. This difference in accumulation is due to the shorter fluorinated carbon chain length and is also dependent on the functional group due to changes in the surfactant properties [36].

The role of specific reactive oxygen species (ROS) in PFOS neurotoxicity has been examined. Superoxide production increases after exposure to PFOS. Mitochondria have also been shown to be decreased in neurons of worms treated with PFOS starting at 5 mg/L (10 µM) [30]. Importantly, the synthetic antioxidant XJB-5-131, which specifically decreases mitochondrial oxidative stress, was able to rescue dopaminergic neurodegeneration and mitochondrial function after PFOS exposure [30]. 

PFOS specifically causes dopaminergic neurotoxicity, starting at 75 mg/L (150 µM) at 48 h, whereas effects on γ-aminobutyric acid (GABA)ergic, serotonergic, and cholinergic neurons did not start until 100 mg/L (200 µM) (Table 2A) [30]. PFOS also caused behavioral defects, such as increased repulsion time, a dopamine-dependent behavior, forward movement, body bending, and thrashing, without changes in paralysis, which is controlled by the cholinergic system (Table 2A) [30,33]. Both the dopaminergic neurons and the behavioral defects were able to be rescued by daily treatment with glutathione, which further indicates ROS as part of the mechanism of toxicity [30]. PFOS has also been implicated in decreased behavioral plasticity, with 20 µM PFOS increasing the chemotaxis index and decreasing the expression of gcy5-gfp in ASE sensory neurons, although it is unclear if this protein is important for chemotaxis in *C. elegans* (Table 2A) [33]. However, these changes in plasticity were not concurrent with clear changes in either cholinergic or dopaminergic neurons at 20 µM PFOS [33]. This data indicates potential deficits in motor function after exposure, but more studies are needed, especially on other PFAS. 

### 3.2. Dugesia japonica

*Dugesia japonica* are flatworms that live in freshwater and are exposed to PFAS mainly through contaminated water, sometimes heavily. *D. japonica* are low on the foodchain, similar to *C. elegans*, so similar precautions must be taken into consideration when extrapolating results to potential human toxicity. Research in *D. japonica* has only focused on the neurotoxicity of PFOS.

Dopamine was decreased in *D. japonica* exposed to 0.5 mg/L (1 µM) PFOS but increased at all doses between 0.5 and 10 mg/L (20 µM) after 1 day of exposure; it was significantly increased on day 4 of all exposed and continued to be increased after 10 days in all doses except 5 mg/L (10 µM) (Table 2A) [45]. Interestingly, this is different than that found in *C. elegans*, where dopamine was decreased after exposure to PFOS [30]. This discrepancy could be due to differences in accumulation between *D. japonica* and *C. elegans*. In *D. japonica*, serotonin was affected after one day of exposure, with 0.5 mg/L PFOS increasing serotonin levels and 1 mg/L decreasing it (Table 2A) [45]. Interestingly, it was decreased at both exposure levels and increased at 5 mg/L and 10 mg/L after 4 days of exposure, while only the 0.5 mg/L and 10 mg/L had significantly increased serotonin after 10 days (Table 2A) [45].

PFOS had differing effects on GABA in *D. japonica*, with an initial decrease after 0.5 mg/L for one day and an increase after 10 mg/L (Table 2A) [45]. Interestingly, after four days of exposure, the 0.5 mg/L group had an increase in GABA while the 5 mg/L group had a decrease, and all exposure groups except the 10 mg/L had a decrease in GABA after 10 days of exposure (Table 2A) [45].

Acetylcholinesterase (AChE) activity was as variable after exposure to PFOS as GABA levels, with activity decreasing after exposure to 0.5 mg/L and 1 mg/L PFOS and increasing at 5 mg/L and 10 mg/L after 1 day of exposure (Table 2A) [45]. This was followed by a decrease in activity in all exposure groups after 3 days except for 10 mg/L, which had a significant increase, with all exposure groups having increased activity after 5 days of exposure (Table 2A) [45]. After 7 days, all but the highest exposure group had increased activity, while the 10 mg/L had decreased activity, and the lowest exposure caused decreased activity after 10 days, while 5 mg/L caused an increase (Table 2A) [45]. These changes suggest different effects of PFOS on the neurotransmitter systems depending on length of exposure and PFOS concentration. Furthermore, there was a dose-dependent decrease in synapsin+ neurons, which indicates a decrease in neurotransmitter synaptic vesicles (Table 2A) [45].

Neuronal development is an area of great concern, especially because the blood–brain barrier is not fully functioning during development [53]. Interestingly, in *D. japonica*, *djotxA (D. japonica* homeobox protein OTX A), a gene that is important for the development of the part of the brain necessary for vision, mRNA was significantly increased at 0.5 mg/L, 1 mg/L, and 5 mg/L PFOS but was decreased after 10 mg/L for 1 and 10 days (Table 2A) [45]. Similarly, other mRNA coding for transcription factors, such as *djotxB* (*D. japonica* homeobox protein OTX B), *djFoxD* (*D. japonica* forkhead box D), *djFoxG*, and *djnlg* (*D. japonica* neuroligin) were all affected by PFOS (Table 2A) [45]. *djotxB* mRNA was increased at 1 mg/L and 5 mg/L after 1 day of exposure, decreased after 10 mg/L exposure on day 4, and decreased in all exposures after 10 days (Table 2A) [45]. *djFoxD* mRNA was significantly decreased at 0.5, 1, and 10 mg/L and increased at 5 mg/L after 1 day of exposure but decreased at all exposures except for 0.5 mg/L after 4 days, and in all treatments after 10 days of exposure (Table 2A) [45]. *djFoxG* mRNA was significantly increased in all PFOS treatments after 1 day, increased at the lowest exposure and decreased with 1 and 10 mg/L after 4 days of exposure, and decreased in all exposure groups after 10 days of treatment (Table 2A) [45]. *djnlg* mRNA was significantly increased at 1 mg/L after the first day of exposure, but it decreased in 1 mg/L and 5 mg/L after four days, and it increased after 10 days of exposure at 0.5 mg/L and 10 mg/L (Table 2A) [45]. These data indicate that PFOS is significantly affecting gene expression, which is important for neurodevelopment in a dose- and time-dependent manner.

## 4. Vertebrate Species Used to Study Neurotoxicity of PFAS

### 4.1. Danio rerio

Fish are exposed to the water that is contaminated by PFAS. Exposure is mostly through gills and diet. Again, this can lead to comparisons, but one must remember that there are differences when considering potential effects on humans. *D. rerio* are good models because they are relatively easy to use in laboratory settings. Development occurs outside of the parent and eggs are clear, which makes them easily manipulated, similar to *C. elegans* [54]. Furthermore, the genetic knowledge we have for them makes them ideal species to study the potential effects of chemicals on gene expression [54]. *D. rerio* have been used abundantly in PFAS research, including studies on PFOS, PFOA, PFBS, PFNA, and more recently found PFAS including both short and long-chain PFAS.

The accumulation of PFAS in *D. rerio* has been studied for many compounds that have not been studied in other species. After 5 days of exposure that started on the day of fertilization, PFOS levels were found to be 15.2 ± 2.4 ng/embryo for 0.5 mg/L PFOS, 52.8 ± 2.1 ng/embryo for 2 mg/L, and 66.1 ± 2.4 ng/embryo for 4 mg/L (Table 1) [46]. For a more acute exposure, embryos exposed for 24 h had 21.6 ± 5.4 ng/mg after 0.1 mg/L and 213.5 ± 62.7 ng/mg after 1 mg/L PFOS [38]. Unfortunately, these are not easily compared due to the measurement of PFOS being per embryo in one study. Interestingly, another study found that the co-exposure to PFOS and single-wall carbon nanotubes decreased the bioaccumulation of PFOS in internal organs, suggesting PFOS interacts with single-wall carbon nanotubes, decreasing the amount readily available for absorption [55]. Studies have also focused on more recently produced PFAS, such as 6:2 fluorotelomer sulfonamide alkylbetaine (6:2 FTAB) and 6:2 fluorotelomer sulfonamide alkylamine (6:2 FTAA). Exposure to 6:2 FTAB for 180 days in fish that were 5 months old at the initiation of exposure caused an accumulation of 6:2 FTAA with no quantifiable amount of 6:2 FTAB present, implying that 6:2 FTAB is readily metabolized with 6:2 FTAA being the main metabolite [56]. Interestingly, females accumulated more 6:2 FTAB in their gonads than males did, but males accumulated more in the liver; the sex difference in accumulation is thought to be due to excretion through spawning and an increased expression of ion transporters in the ovaries [56]. Furthermore, when parents were exposed to 6:2 FTAB prior to spawning, the F1 generation also had dose-dependent levels present, indicating that there is transfer from parents to offspring [56]. While accumulation has been studied fairly well for PFOS and some of the more recent PFAS, there is still a lack of accumulation data for the brains of *D. rerio* or for other PFAS, such as PFOA and other shorter chain PFAS.

Behavioral deficits have been a prominent area of study in *D. rerio* exposed to PFAS because behavioral changes can indicate effects on different aspects of the nervous system. In some studies, embryos exposed to 0.5 mg/L PFOS through 96 h post fertilization (hpf) or through 144 hpf at 0.03–12 mg/L swam significantly faster than controls (Table 2A) [46,49]. Interestingly, another study that exposed embryos through 120 hpf at 0.02 µM found that PFOS decreased swimming speed while PFOA increased it (Table 2A) [47]. Therefore, the effects of PFOS on swimming speed could change based on level of exposure, as higher concentrations caused increased speed, while lower concentrations caused decreased speed. These differential effects could be common among PFAS, as PFNA had similar discrepancies, with one study showing decreased speed after exposure to 0.02–2 µM PFNA through 120 hpf and then reared to 30 days post fertilization (dpf), but another showing increased speed in males specifically after the same exposure time at 2 µM, indicating potential sex differences (Table 2B) [47,48]. Perfluorododecanoate (PFDoA) also decreased the average swimming, speed starting at 0.24 mg/L PFDoA through 120 hpf (Table 3) [51].

Interestingly, effects on speed and the amount of movement do not tend to correlate after exposure to PFAS. PFOS (>1.5 mg/L), PFBS (>450 mg/L), PFNA (>16 mg/L), and trifluoroacetate (TFAA) (>700 mg/L) all decreased movement during developmental exposure until 144 hpf (Table 2) [49]. Furthermore, another study found that 1 mg/L PFOS through 6 dpf also decreased bouts of swimming, although it increased total movement (Table 2A) [38]. The same study found that co-exposure to dexamphetamine, a catecholamine reuptake inhibitor, or the dopamine receptor agonists quinipirole (D1) or SKF-81297 (D2) was able to rescue the decreased movement due to PFOS [38]. This indicates that PFOS could be affecting the motor system through dopamine, but more research is necessary to determine how PFOS is affecting it specifically. Interestingly, PFNA also decreased swimming after exposure through 120 hpf and then rearing to 30 dpf, indicating that decreased swimming could be a trend among PFAS (Table 2B) [48]. Interestingly, PFOS, PFOA, and PFNA all increased the distance swam in the dark after exposure through 120 hpf followed by 2 weeks of depuration (Table 2) [47]. The hyperactivity after PFOS exposure was corroborated in a study where *D. rerio* exposed through 6 dpf had significantly increased movement, but exposure to PFOA did not affect movement [57]. Furthermore, 89 µM perfluoroheptanoate (PFHpA) also increased the swimming distance in the light cycle after exposure through 144 hpf (Table 3) [58]. Both PFHxS and PFHxA caused increased activity along with perfluoropentane sulfonate (PFPeS) and PFHpS [57]. However, the PFAS 1H-indole-2-sulfonic acid, 5-((aminocarbonyl)hydrazono)-2,3,5,6-tetrahydro-1-methyl-6-oxo salt (ADONA), PFBS, and perfluoro-3,6-dioxa-4-methyl-7-octene-1-sulfonate (PFESA1) did not have any effects on behavior [57]. These data on swimming speed and distance indicate that various PFAS could be affecting various systems, such as motor or anxiety, which should be studied in other models as a potential effect of PFAS exposure.

Another area of neurodevelopment studied is the visual motor response, where fish generally increase activity at the beginning of the dark cycle and then slow down. Fish exposed to 1 mg/L PFOS through 6 dpf did not respond to dark stimulation, while fish exposed to 0.1 mg/L had decreased movement in general during the dark phase (Table 2A) [38]. Interestingly, these effects were also rescued with exposure to dexamphetamine, quinpirole, or SKF-81297 [38]. Exposure to PFDoA through 120 hpf had different effects after light phase changes, which included increased time to slow down after the lights turned on and increased time to speed up when lights were shut off, indicating potential effects on the visual motor response (Table 3) [51]. Overall, these studies show that developmental exposure to PFOS or PFDoA affects the visual motor response, although potentially in different ways depending on the chemical and the concentration, and that this should be further studied in other systems.

There are many indicators in *D. rerio* that can be indicative of escape behavior, the fight-or-flight response, or anxiety. One of the most common studied is burst movements, which are fast, large movements for short periods of time [59]. Interestingly, PFOA, PFNA, PFOS, and a mixture of PFHxA, PFHpA, PFOA, PFNA, PFBS, PFHxS, PFOS, and perfluoropentanoate (PFPeA) exposure through 144 hpf all increased burst movements during the dark phase, while PFHxS and PFOS were the only PFAS tested that increased burst movements during the light phase (Table 2) [58].

Other ways to determine anxiety behaviors are through measuring the time spent in the middle of a dish, time freezing, or the response to a startle [59]. One study found that 2 µM PFNA exposure through 120 hpf and then 2 weeks of depuration led to increased time in the middle, indicating less anxiety, while the time did not change with PFOS or PFOA exposure (Table 2) [47]. Interestingly, in another study where fish were exposed to PFAS through 120 hpf and then reared to 6 months, 2 µM PFNA exposed males spent less time in the middle of the tank, while PFOS and PFOA-exposed males did not have significant changes (Table 2) [48]. Interestingly, in the same study, males exposed to PFNA had decreased time of immobility, potentially further indicating decreased anxious behavior or increased reckless behavior (Table 2B) [48]. In another study, 1 mg/L PFOS through 6 dpf caused an increased startle response and increased bouts of burst movement after startle, indicating that PFOS exposure could be increasing anxiety (Table 2A) [38]. Interestingly, PFNA exposure also caused males to attack their reflection more, indicating more aggression, while PFOS exposure decreased attacks (Table 2A,B) [48]. Importantly, while the findings should be confirmed in mammalian models, it is worth noting that schizophrenia risk genes are well conserved across taxa and fish models have been highly useful in the study of chemically induced phenotypes relevant to psychiatric disorders [60,61,62]. Thus, these data indicate that PFAS could be causing neurological effects and should be studied further as potentially increasing risk of psychiatric disorders.

As for neurotransmitter levels, data are lacking on effects of exposure. It has been shown that PFOS did not affect AChE activity in the liver, intestine, or gills; however, activity was not quantified in the brain, and co-exposure with single-walled carbon nanotubes increased activity (Table 2A) [55]. However, exposure to 6 mg/L PFDoA through 120 hpf decreased the total acetylcholine in the body, while both 1.2 and 6 mg/L PFDoA decreased the AChE activity and mRNA expression (Table 3) [51]. This could indicate that PFDoA is also decreasing the formation of acetylcholine, leading to the decreased accumulation of acetylcholine and activity of AChE, although this has to be further studied with a focus on the brain. Interestingly, both 1.2 and 6 mg/L PFDoA exposure also increased dopamine levels, which is the opposite of what has been shown with PFOS in *C. elegans* (Table 3) [51]. Interestingly, PFDoA exposure decreased the expression of *syn2a* (synapsin IIa), which is a protein that plays a role in serotonin and dopamine release (Table 3) [51]. While this could decrease the release of dopamine, PFDoA also increases *manf* (mesencephalic astrocyte-derived neurotrophic factor) expression, which plays a role in dopaminergic neuron survival (Table 3) [51].

Furthermore, PFAS exposure causes the differential expression of genes important for neurodevelopment and neuronal function. PFOS increases α-tubulin protein in motor neurons of the spinal cord with no changes in the brain after 96 h of exposure, but it decreases expression in both the spinal cord and brain after 120 h (Table 2A) [63]. Interestingly, 6 mg/L of PFDoA exposure through 120 hpf decreased *α1-tubulin* gene expression in the brain as well, indicating that longer developmental exposures to PFAS could decrease tubulin in the brain (Table 3) [51]. PFOS also affected DNA replication, which is indicated by proliferating cell nuclear antigen (*pcna*) gene expression decreasing after 24 h and increasing after 96 and 120 h, implying decreased DNA replication at early time points and increased DNA replication at later time points (Table 2A) [63]. The neurogenesis-regulating gene *cdk5* (cyclin-dependent protein kinase 5) was also increased at the later time points after PFOS exposure, corroborating the idea that neurogenesis occurs later in PFOS-exposed *D. rerio* (Table 2A) [63]. However, PFDoA exposure through 120 hpf shows opposite results, such as decreased *gap43* (growth-associated protein 43), *shha* (sonic hedgehog protein A), and *Elavl3* (ELAV like RNA binding protein 3), which are all important for neurogenesis, and decreased fluorescence of GFP expressed through the promoter for *elavl3*, which is commonly used as a neuronal marker (Table 3) [51]. Interestingly, it also decreases *gfap* (glial fibrillary acidic protein), potentially indicating decreased astrocytes, and *mbp* (myelin basic protein) (Table 3) [51].

### 4.2. Oryzias melastigma

The main reasons for using fish as sentinel species is discussed in Section 4.1, although most fish are not used in the laboratory setting, such as *D. rerio*. However, when studying other types of fish, you must consider their place in the food chain. Marine medaka (*Oryzias melastigma*) are relatively low on the food chain, eating zooplankton and phytoplankton. These animals still have similar systems to humans, but their accumulation and effects could be different than humans and should be taken into consideration. Unfortunately, the only neurotoxicology studies in *O. melastigma* is on PFBS.

Both norepinephrine and epinephrine were increased in female *O. melastigma* exposed to 9.5 µg/L PFBS, but males had significantly decreased epinephrine after the same exposure (Table 3) [50]. There were no changes in serotonin after exposure to PFBS, though, indicating that toxicity is specific to certain neurotransmitters (Table 3) [50].

Acetylcholine levels were increased in male *O. melastigma* exposed to 1 µg/L (3.33 nM) PFBS, although there were not any changes in AChE activity or choline, which are the enzymes that metabolize acetylcholine to choline and acetylcholine’s major metabolite, respectively (Table 3) [50]. Interestingly, acetylcholine levels were increased in both 1 and 9.5 µg/L (31 nM) PFBS in female *O. melastigma*, while choline was significantly increased at the higher exposure without changes in AChE activity (Table 3) [50]. This further indicates that the cholinergic system function should be studied as potential areas of concern with exposure to PFBS.

Again, neuronal development is an area of great concern, especially because the blood–brain barrier is not fully functioning during development [53]. Studies in *O. melastigma* found that both males and females exposed during development had decreased ratios of eye-to-body weight and increased water in the eye after 1 µg/L PFBS for males and 9.5 µg/L for females, and that PFBS accumulated in the eye in a dose-dependent manner [50]. Both β- and γ-crystallin, proteins important for many functions within the eye, were decreased in male *O. melastigma* exposed to 9.5 µg/L PFBS, while in females, only γ-crystallin was significantly down-regulated (Table 3) [50]. Male *O. melastigma* also had decreased arrestin, which is a G-protein coupled receptor that is important for the visual rhodopsin system, and increased lumican, which is a proteoglycan that is important for maintaining transparency of the eye, after exposure to PFBS (Table 3) [50]. These data indicate that PFBS is affecting the eye, potentially indicating toxicity in other organisms in a similar manner.

### 4.3. Frogs

Frogs are common species used for studying toxicology, especially chemicals that are found in the environment, such as PFAS. The types of frogs used for studying toxicology vary greatly. *Xenopus* are commonly used in laboratories because of their fully known genetic makeup and ease of use in the laboratory, including breeding [64]. However, *Xenopus* are not very closely related to other frogs, so extrapolation of data is difficult [64]. One of the advantages to using frogs as model systems is that, while their anatomy is not as closely related as mammals, they have similar systems to humans, and certain frogs, specifically Northern leopard frogs (*Rana pipiens*), contain neuromelanin, which is a conglomeration of lipids, proteins, and oxidized dopamine, similar to humans, whereas lower order mammals, such as rats and mice, do not produce this to as large an extent as humans [65]. The neurotoxicity of PFOA and PFOS has been studied in *R. pipiens*.

Exposure to PFOS or PFOA caused an accumulation of 4500 ppb (ng/g) PFOS and 169 ppb (ng/g) PFOA in *R. pipiens* exposed to 1000 µg/L PFOS or PFOA, with PFOS increasing in a dose-dependent manner while PFOA did not change based on exposure level (Table 1) [39]. American bullfrogs (*Lithobates catesbeianus*) have a calculated LC_50_ of 144 mg/L (288 µM) PFOS and 1004 mg/L (2.42 mM) PFOA, which is much higher than for *C. elegans* [66].

Little has been studied on the effects of PFAS on the brain of frogs and how it affects neurotransmitter systems. We previously showed that 100 and 1000 µg/L PFOS and 1000 µg/L PFOA were able to significantly decrease dopamine levels in whole brain of *R. pipiens*, with a significant increase in dopamine turnover at 1000 µg/L for both chemicals (Table 2A,C) [39]. Dopamine was the only neurotransmitter affected in this study, indicating that exposures of PFOS or PFOA could lead to specifically dopaminergic degeneration, similar to *C. elegans*, although more studies need to be performed to determine effects at more environmentally relevant levels and to determine the specific systems being affected.

### 4.4. Ursus maritimus

Aquatic and semiaquatic mammals such as *Ursus maritimus* have been proposed as leading sentinel species [67]. This is partially due to their similar physiology to humans, being mammals. Other factors are that they have long lives, allowing for lifetime exposure similar to humans, and that they are normally high on their food chain [67]. They also are thought to accumulate chemicals in a manner more similar to humans, and they are proposed to have similar effects with chemical exposure through routes such as lactation, as humans do [67]. It is also thought that they might show signs of toxicity from chemicals more strongly than other sentinel species, making them better to make clear the necessity for further research and working toward solutions [67]. Research on *U. maritimus* has been performed on bears that were in the wild, so exposure was to all PFAS in the environment.

*U. maritimus* in East Greenland had liver levels of PFAS averaging 3546 ng/g wet weight while male *U. maritimus* had an average of 22.92 ng/g wet weight PFOS, 28.82 ng/g total perfluorosulfonates, 1.09 ng/g PFOA, and 99.40 ng/g total perfluorocarboxylates in the brain (Table 1) [42,52]. Another study on *U. maritimus* mothers found plasma levels of total PFAS to be 539.0 ± 20.8 ng/g wet weight, with PFOS and PFOA levels equaling 431.9 ± 17.0 ng/g wet weight and 6.4 ± 0.6 ng/g wet weight [68].

Another study on *U. maritimus* from East Greenland studied the effects on various parts of the brain [52]. Glutathione synthase activity was positively correlated with levels of PFOS, total perfluorosulfonates, and perfluoroundecanoate (PFUnDA) and borderline correlated to levels of perfluorododecanoate (PFDoDA), perfluorotetradecanoate (PFTrDA), and total perfluorocarboxylates in the occipital lobe, and positively correlated with PFOS and total perfluorosulfonates in the frontal cortex while being negatively correlated with total perfluorosulfonates in the hypothalamus (Table 3) [52]. This indicates that there is generally an upregulation of oxidative stress mechanisms with increased levels of PFAS in the brain. This study also found that monoamine oxidase activity, one of the main enzymes that metabolizes catacholamines such as dopamine, serotonin, and norepinephrine, was positively correlated with levels of PFTrDA, total perflurocarboxylates, and trended to correlate with PFDoDA in the occipital lobe and with levels of PFOS, total perfluorosulfonates, PFUnDA, PFDoDA, and total perfluorocarboxylates in the whole brain (Table 3) [52]. There was also a negative correlation between the density of dopamine D2 receptors and PFUnDA, PFDoDA, PFTrDA, and total perfluorocarboxylates in the temporal cortex and a borderline negative correlation with PFTrDA and total perfluorocarboxylates in the cerebellum (Table 3) [52].

The density of GABA-A receptors was also positively correlated with PFOS and PFDoDA levels and borderline correlated with total perfluorosulfonate levels, PFUnDA, and total perfluorocarboxylate levels across the brain (Table 3) [52]. Furthermore, there was a negative correlation between muscarinic acetylcholine receptor density and PFUnDA, PFTrDA, and total perfluorocarboxylates that was also borderline for PFOS and total perfluorosulfonates in the cerebellum (Table 3) [52]. This was accompanied by a negative correlation between AChE activity and PFDoDA and PFUnDA in the cerebellum (Table 3) [52]. Therefore, PFAS are affecting cerebellar cholinergic transmission, which is similar to that seen in fish and *C. elegans*. There was also a negative association between AChE activity and PFDoDA in the thalamus and with PFTrDA and total perfluorocarboxylates in the frontal cortex (Table 3) [52]. Muscarinic acetylcholine receptor density was also positively correlated with PFOS and total perfluorosulfonate levels in the frontal cortex (Table 3) [52]. The difference in correlations with muscarinic acetylcholine receptors and PFOS between the cerebellum and the frontal cortex implies that there might be different reactions to PFOS and other perfluorosulfonates in different parts of the brain.

## 5. Critical Analyses of the Use of Sentinels in PFAS Toxicity: Integration of Findings across Sentinel and Non-Traditional Models and Potential to Predict Adverse Outcomes

As for how these data relate to each other, it is important to look across all the species discussed to determine the translational value and potential findings from discrepancies. The neurological effects of PFAS are poorly understood thus far, but all studies on the brains of exposed animals showed some form of change. Furthermore, there are few studies determining the effects of PFAS other than PFOS and PFOA, with only a few studying neuronal changes after shorter chain PFAS exposure such as PFBS, PFBA, and TFAA, or longer chain PFAS such as PFDA and PFDoA. Currently, there are two consistent results. PFAS appear to affect dopamine neurotransmission, as *C. elegans* and frogs had decreased dopamine and *U. maritimus* with higher levels of PFAS had higher activity of monoamine oxidase, potentially indicating a higher rate of metabolism of neurotransmitters such as dopamine. This is similar to the finding that male mice exposed to PFOS during development had decreased motor function including decreased movement and decreased latency to fall on the rotarod, where dopamine is one of the main neurotransmitters necessary for motor function [69]. Other studies, such as in fish, *D. japonica*, and *U. maritimus*, have also implicated changes in acetylcholine levels, with acetylcholine increasing in exposed fish and AChE activity decreasing in *U. maritimus* with higher PFAS levels. However, studies with fish have also shown changes in neurotransmitters differing depending on time and exposure level. These differences could be due to different levels of accumulation and a time-based response; however, further research needs to be performed to determine these possibilities. Differences in accumulation is also important to remember when comparing different species, such as the different effects on *C. elegans* and *D. japonica* compared to fish, which could be due to differences in accumulation. Therefore, it is probable that PFAS are affecting neurotransmitter levels, especially dopamine and acetylcholine. Further tests should be performed in model systems to determine the potential effects on motor function and memory, which are two important functions controlled by dopaminergic and cholinergic systems.

Unfortunately, the majority of testing on sentinel species and non-traditional laboratory models has not included the quantification of PFAS levels in the blood and/or brain tissue after exposure. This complicates the ability to directly compare toxicities across species because differences in toxicokinetics will influence PFAS target organ concentrations and ultimately, effects. Comparing toxicities of PFAS among animals that have similar accumulation levels would be more indicative of whether these compounds are affecting different species in similar ways. This is clear when comparing levels of PFOS in *C. elegans*, *D. rerio*, and *R. pipiens*, where it is apparent that *C. elegans* accumulate PFOS more readily than frogs, while *D. rerio* accumulate PFOS to a lower extent than *R. pipiens*.

Furthermore, the specific PFAS studied for neurotixicity effects is very limited for the majority of organisms. Research on the neurological effects of PFAS other than PFOS and PFOA is necessary to determine differences in the toxicity of these compounds. Studies in *D. rerio* and *U. maritimus* indicate that shorter chain PFAS also affect the brain. However, research on the short-chain PFAS tend to be lacking in sentinel and non-traditional model organisms.

## 6. Conclusions

The accumulation of PFAS is an important aspect of potential toxicity. Humans have been found to accumulate PFOS up to 3490 ng/mL in a retired flurochemical worker [7]. Furthermore, livers from human cadavers had 4 ng/g wet weight PFOS and 0.7 ng/g wet weight PFOA [70]. Interestingly, both PFOS and PFOA were also present in the brain at measurable levels, with 0.37 ng/g wet weight PFOS and 0.2 ng/g wet weight PFOA [70]. Unfortunately, there are few studies that have measured the accumulation of PFAS in various species. Furthermore, the toxicokinetics of PFAS is an aspect that is poorly understood in most of these non-traditional model systems. Therefore, it is important to understand accumulation and excretion, as these could affect the toxicity seen in different models. Comparison of the accumulation could allow for better studies to find potential effects that might be present in humans at the levels we accumulate.

Importantly, no correlation was found between PFOS or PFOA levels in infants with time to reach developmental milestones such as when the infant started walking on its own or drinking from a cup [20]. These potentially could indicate no effects on neurodevelopment, which could affect motor function necessary for walking or drinking; however, there is little research performed specifically on neurotoxic effects, which needs further work to determine potential effects. Furthermore, there have been no studies on PFAS exposure levels and psychiatric disorders or the risk of neurodegenerative diseases later in life, such as Parkinson’s disease, as research indicates potential deleterious effects on dopaminergic systems.

Overall, it appears that there are mostly consistent findings on the effects of PFAS on sentinel and non-traditional model species. However, much of the work presented here is the first of its kind, so it is imperative that research continues to corroborate the findings thus far and help elucidate what is occurring when there are varied results from study to study. It is also imperative to study the effects of different PFAS, as it appears that there are differences in effects due to chain length and functional group.

## Figures and Tables

**Table 1:** Examples of PFOS concentrations in different sentinel and non-traditional laboratory model species.

Species	Sample Type	PFOS (µg/mg)	Exposure	Exposure Time	Reference
*C. elegans*	Whole body	13.06	1 mg/L	72 h	Sammi, 2019 [30]
*Oreochromis mossambicus*	Whole body	0.0000416	n/a	n/a	Bangma, 2017 [37]
*D. rerio*	Whole body	0.000021.6	1 mg/L	6 days	Spulber, 2014 [38]
*R. pipiens*	Whole body	0.0045	1 mg/L	30 days	Foguth, 2019 [39]
*Tursiops truncatus*	Plasma	0.000571	n/a	n/a	Soloff, 2017 [40]
*Pusa hispida*	Serum	57.3 ng/mL	n/a	n/a	Levin, 2016 [41]
*U. maritimus*	Liver	0.00002882	n/a	n/a	Biosvert, 2019 [42]
*Tachycineta bicolor*	Serum	137 ng/mL	n/a	n/a	Custer, 2012 [43]
*Sus scrofa*	Liver	0.000040	n/a	n/a	Watanabe, 2010 [44]

**Table 2:** Neurotoxicity endpoints and findings in species where neurological endpoints were tested. (**A**). PFOS neurotoxicity and findings in species where neurological endpoints were tested (**B**). PFNA neurotoxicity endpoints and findings in species where neurological endpoints were tested. NT = not tested (**C**). PFOA neurotoxicity endpoints and findings in species where neurological endpoints were tested. NT = not tested. Δ—indicates a change after exposure that is not consistent over dose or time, i.e., nonmonotonic dose response.

**(A)**						
**Organism**	**Concentration (µM)**	**Length of Exposure**	**Neurobehavior**	**Neurotransmitters**	**Neuropathology**	**Reference**
*C. elegans*	40–400	72 h	↑Repulsion time	NT	↓Dopaminergic neurons	Sammi, 2019 [30]
*C. elegans*	20	48 h	↑Forward movement and thrashing	NT	↓Dopaminergic and cholinergic neurons	Chen, 2014 [33]
*D. japonica*	1–20	5–7 d	NT	↑Dopamine Δ Serotonin Δ GABA	Δ Acetylcholinesterase activity Δ Neurodevelopmental genes	Yuan, 2018 [45]
*D. rerio*	2	6 d	↓Bouts ↑Distance during bout ↑Reaction to light changes ↑Startle	NT	NT	Spulber, 2014 [38]
*D. rerio*	1–8	1–114 h	Δ Speed	NT	NT	Huang, 2010 [46]
*D. rerio*	0.02–2	14 d	↑Distance Speed	NT	NT	Jantzen, 2016 [47]
*D. rerio*	2 µM	117 h, 6 m depuration	↓Hitting glass-males	NT	NT	Jantzen, 2016 [48]
*D. rerio*	0.06–20	144 h	Δ Activity ΔTime active	NT	NT	Ulhaq, 2013 [49]
*R. pipiens*	0.2–2	30 d	NT	↓Dopamine ↑Dopamine turnover	NT	Foguth, 2019 [39]
(**B**)						
**Organism**	**Concentration (µM)**	**Length of Exposure**	**Neurobehavior**	**Neurotransmitters**	**Neuropathology**	**Reference**
*D. rerio*	0.02–1	14 d	↓Distance ↓Speed	NT	NT	Jantzen, 2016 [47]
*D. rerio*	2	117 h, 6 m depuration	↓Distance ↓Time in the middle ↓Time frozen ↑Speed ↑Hitting glass-males ↑Time in light-males	NT	NT	Jantzen, 2016 [48]
*D. rerio*	0.06–22	144 h	Δ Activity Δ Time active	NT	NT	Ulhaq, 2013 [49]
(**C**)						
**Organism**	**Concentration (µM)**	**Length of Exposure**	**Neurobehavior**	**Neurotransmitters**	**Neuropathology**	**Reference**
*D. rerio*	0.2	14 d	↑Distance	NT	NT	Jantzen, 2016 [47]
*D. rerio*	2	117 h, 6 m depuration	↓Time in light-females	NT	NT	Jantzen, 2016 [48]
*D. rerio*	7.2–2415	144 h	Δ activity Δtime active	NT	NT	Ulhaq, 2013 [49]
*R. pipiens*	2.4	30 d	NT	↓Dopamine ↑Dopamine turnover	NT	Foguth, 2019 [39]

**Table 3:** Other PFAS neurotoxicity endpoints and findings in species where neurological endpoints were tested. NT = not tested. Δ—indicates a change after exposure that is not consistent over dose or time, i.e., nonmonotonic dose response.

Organism	Chemical	Concentration (µM)	Length of Exposure	Neurobehavior	Neurotransmitters	Neuropathology	Reference
*O. melastigma*	PFBS	0.03	6 m	NT	↑Dopamine Sex-specific Δ norepinephrine Δ Serotonin over time Δ GABA over time ↑Acetylcholine	Δ Transcription factors involved in visual development	Chen, 2018 [50]
*D. rerio*	TFAA, PFBA, PFDA (perfluorodecanoate), or PFBS	48–14, 33.3–10000, 0.2–58.4, or 33.3–10000	144 h	Δ Activity ΔTime active	NT	NT	Ulhaq, 2013 [49]
*D. rerio*	PFDoA	0.4–10	120 h	↓Speed	↓Acetylcholine ↑Dopamine	↓Acetylcholinesterase	Guo, 2018 [51]
*U. maritimus*	PFBS, PFHxS, PFOS, perfluorodecane sulfonate (PFDS), PFHxA, perfluoroheptanoate (PFHpA), PFOA, PFNA, PFDA, perfluoroundecanoate (PFUnDA), perfluorododecanoate (PFDoDA), perfluorotridecanoate (PFTrDA), perfluorotetradecanoate (PFTeDA), and perfluoropentadecanoate (PFPeDA) were quantified. PFAS levels were due to exposure in the wild	PFBS: 0.55 ± 0.08 PFHxS: 1.10 ± 0.10 PFOS: 22.92 ± 0.84 PFDS: 0.66 ± 0.06 PFHxA: 0.13 ± 0.03 PFHpA: not detected PFOA: 1.09 ± 0.13 PFNA: 2.59 ± 0.13 PFDA: 2.63 ± 0.15 PFUnDA: 22.30 ± 1.14 PFDoDA: 8.19 ± 0.46 PFTrDA: 37.87 ± 2.29 PFTeDA: 6.81 ± 0.40 PFPeDA: 4.71 ± 0.42 ng/g wet weight in the whole brain	Unknkown, Ages of bears at sampling were 2–10 years	NT	NT	↑Glutathione synthase in occipital lobe and frontal cortex ↓Glutathione synthase in hypothalamus ↑Monoamine oxidase activity ↓Dopamine D2 receptors in occipital lobe and cerebellum ↓Muscarinic acetylcholine receptor activity in cerebellum ↑GABA-A receptors ↑Muscarinic acetylcholine receptor activity in frontal cortex ↓Acetylcholinesterase activity in frontal cortex	Eggers Pederson, 2015 [52]
